# The Care of the Eyes

**Published:** 1879-02-20

**Authors:** 


### *THE CARE OF THE EYES.*

All are anxious to do this, but few know how effectually to do so, and many never think of the matter till failing eye-sight warns them that it is absolutely necessary. By the latter the following suggestions will be read with interest.

The sight in most persons begins to fail from forty to fifty years of age, as is evidenced by an instinctive preference of large print; a seat near the window for reading is selected; there is an effort to place the paper at a convenient distance from the eye, or to turn it so as to get a particular reflection of the light; next the finger begins to be placed under the line read, and there is a winking of the eye as if to clear it, or a looking away at some distant object to rest it; or the fingers are pressed over the closed lids in the direction of the nose, to remove the surplus tears caused by straining.

Favor the failing sight as much as possible. Looking into a bright fire, especially a coal fire, is very injurious to the eyes. Looking at molten iron will soon destroy the sight; reading in the twilight is injurious to the eyes, as they are obliged to make great exertion. Reading or sewing with a side light injures the eye, as both eyes should be exposed to an equal degree of light. The reason is, the sympathy between the eyes is so great that if the pupil of one is dilated by being kept partially in the shade, the one that is most exposed cannot contract itself sufficiently for protection, and will ultimately be injured. Those who wish to preserve their sight should observe the following rules, and preserve their general health by correct habit:

1st. By sitting in such a position as will allow the light to fall obliquely over the shoulder upon the page or sewing.

2d. By not using the eyes for such purposes by any artificial light.

3d. By avoiding the special use of the eyes in the morning before breakfast.

4th. By resting them for half a minute or so, while reading or sewing, or looking at small objects; and by looking at things at a distance, or up to the sky; relief is immediately felt by so doing.

5th. Never pick any collected matter from the eyelashes or corners of the eyes with the finger-nails; rather moisten it with the saliva and rub it away with the ball of the finger.

6th. Frequently pass the ball of the finger over the closed eyelids towards the nose; this carries off any excess of water into the nose itself by means of the little canal which leads into the nostrils from each inner corner of the eye, this canal having a tendency to close up in consequence of the slight inflammation which attends weakness of the eyes.

7th. Keep the feet always dry and warm, so as to draw any excess of blood from the other end of the body.

8th. Use eye-glasses at first carried in the vest-pocket attached to a guard, for they are instantly adjusted to the eye with very little trouble, whereas, if common spectacles are used, such a process is required to get them ready, that to save trouble, the eyes are often strained to answer a purpose.

9th. Wash the eyes abundantly every morning. If cold water is used, let it be flapped against the closed eyes with the fingers, not striking hard against the balls of the eyes.

10th. The moment the eyes feel tired; the very moment you are conscious of an effort to read or sew, lay aside the book or needle, and take a walk for an hour, or employ yourself in some active exercise not requiring the close use of the eyes.—*Monthly Magazine of Pharmacy.*

